# Phenanthrenes from *Juncus Compressus* Jacq. with Promising Antiproliferative and Anti-HSV-2 Activities

**DOI:** 10.3390/molecules23082085

**Published:** 2018-08-20

**Authors:** Csaba Bús, Norbert Kúsz, Gusztáv Jakab, Seyyed Ashkan Senobar Tahaei, István Zupkó, Valéria Endrész, Anita Bogdanov, Katalin Burián, Boglárka Csupor-Löffler, Judit Hohmann, Andrea Vasas

**Affiliations:** 1 Department of Pharmacognosy, University of Szeged, 6720 Szeged, Hungary; bcsaba0312@gmail.com (C.B.); kusznorbert@gmail.com (N.K.); csupor.boglarka@pharmacognosy.hu (B.C.-L.); hohmann@pharm.u-szeged.hu (J.H.); 2 Institute of Environmental Sciences, Faculty of Water and Environmental Management, Szent István University Szarvas, H-5540 Szarvas, Hungary; cembra@freemail.hu; 3 Department of Pharmacodynamics and Biopharmacy, University of Szeged, 6720 Szeged, Hungary; ashkan.tahaei@pharm.u-szeged.hu (S.A.S.T.); zupko@pharm.u-szeged.hu (I.Z.); 4 Interdisciplinary Centre of Natural Products, University of Szeged, 6720 Szeged, Hungary; 5 Department of Medical Microbiology and Immunobiology, University of Szeged, 6720 Szeged, Hungary; endresz.valeria@med.u-szeged.hu (V.E.); bogdanov.anita@gmail.com (A.B.); burian.katalin@med.u-szeged.hu (K.B.)

**Keywords:** *Juncus compressus*, phenanthrene, flavonoid, anti-HSV-2, antiproliferative

## Abstract

Juncaceae species are rich sources of phenanthrenes. The present study has focused on the isolation and structure determination of biologically active components from *Juncus compressus*. Eleven compounds (nine phenanthrenes and two flavonoids) have been isolated from the plant by the combination of different chromatographic methods. Two compounds (compressins A (Compound **1**) and B (Compound **2**)) are novel natural products, while seven phenanthrenes (effusol (Compound **3**), effususol (Compound **4**), juncusol (Compound **5**), 2-hydroxy-1-methyl-4-oxymethylene-5-vinyl-9,10-dihydrophenanthrene (Compound **6**), 7-hydroxy-1-methyl-2-methoxy-5-vinyl-9,10-dihydrophenanthrene (Compound **7**), effususin A (Compound **8**), and dehydroeffusol (Compound **9**)), and two flavonoids (apigenin (Compound **10**) and luteolin (Compound **11**) were isolated for the first time from the plant. Compressin B (Compound **2**) is a dimeric phenanthrene, in which two juncusol monomers (Compound **5**) are connecting through their C-3 atoms. The structure elucidation of the isolated compounds was carried out using 1D, 2D NMR spectroscopic methods and HR-MS measurements. In vitro investigation of the antiproliferative effect of the phenanthrenes on two cervical (HeLa and SiHa) and an ovarian human tumor cell line (A2780) revealed that compounds have remarkable antiproliferative activity, mainly on the HeLa cell line. Moreover, juncusol (Compound **5**) proved to possess significant antiviral activity against the herpes simplex 2 virus (HSV-2).

## 1. Introduction

During the last few decades, several *Juncus* species have been chemically characterized, and the members of this genus have been reported to contain numerous types of natural compounds, including flavonoids [1], coumarins [2], terpenes [3], steroids [4], phenolic acid derivatives [5], stilbenes [6], and a dihydrodibenzoxepin [7]. However, according to the literature data, the most characteristic compounds of these species are phenanthrenes [8]. The occurrence of phenanthrenes is quite rare; only a few plant families accumulate such compounds, and the most important sources of phenanthrenes are species belonging to Orchidaceae, Dioscoreaceae, Betulaceae, Hepaticae and Juncaceae families [9].

In the past few decades, phenanthrenes have become of great interest from phytochemical and pharmacological points of view. To date, up to 90 phenanthrenes were isolated from Juncaceae species (*Juncus acutus*, *J. effusus*, *J. inflexus*, *J. maritimus*, *J. roemerianus*, *J. setchuensis*, *J. subulatus*, and *Luzula luzuloides*) [8]. These compounds are important chemotaxonomic markers, as the presence of a vinyl group in the molecule is characteristic only for Juncaceae phenanthrenes [8,9]. The most important pharmacological effects of phenanthrenes are the antiproliferative, anti-inflammatory, antioxidant, antimicrobial, spasmolytic and anxiolytic activities [9,10].

In the past few years several phenanthrenes, isolated from the members of the genus *Juncus*, were tested for their in vitro cytotoxicity against various cancer cell lines in different test systems. Many of them possessed promising activity; e.g., 2,7-dihydroxy-1-methyl-5-aldehyde-9,10-dihydrophenanthrene exhibited cytotoxic activity (9.17 μM against MCF-7 cells, and 19.6 μM against HeLa cells, respectively) compared to adriamycin (0.406 µM (MCF-7) and 0.539 µM (HeLa)) [11]. The phenanthrene 5-(1-methoxyethyl)-1-methyl-phenanthren-2,7-diol possessed selective inhibitory activity (IC_50_ 10.9 µM) on MCF-7 cells. Dehydroeffusal inhibited the growth of HepG2 (liver hepatocellular carcinoma) and HeLa cells with similar IC_50_ values (12.4 and 13.1 µM, respectively) [12]. Dehydroeffusol inhibited the proliferation and migration of SGC-7901 and AGS gastric cancer cell lines in vitro and exhibited significant suppression of SGC-7901 cell-mediated vasculogenic mimicry in vitro and in vivo without substantial acute toxicity [13]. It was also observed that this compound effectively inhibited the gastric cell growth and the tumorigenicity through inducing tumor suppressive endoplasmic reticulum (ER) stress responses and concurrently diminishing tumor adaptive ER responses [14]. The dimeric phenanthrene effususin B possessed pronounced cytotoxic activity against HepG2 (IC_50_ 12.9 μM), MCF-7 (IC_50_ 12.5 μM) and SMMC-7721 (IC_50_ 13.6 μM) cell lines compared to paclitaxel (36.8 μM (HepG2), 28.6 μM (MCF-7), and 0.09 μM (SMMC-7721)) as a positive control in the CCK-8 assay [10].

To date, only a few phenanthrenes were tested for their antimicrobial activities. Among them, juncusol inhibited *Bacillus* species at all concentrations, while *Planococcus* species were inhibited only at the highest concentration [15]. Dehydroeffusol was proved to be active against methicillin-susceptible and -resistant *Staphylococcus aureus* (MSSA and MRSA), *Bacillus subtilis* and *Candida albicans* in normal (dark) and UVA irradiated conditions. It was observed that under UVA irradiation, dehydroeffusol functioned as a DNA-binding photosensitizer. It strongly inhibited those restriction enzymes (*Kpn*I, *Xba*I, *Pme*I, *Dra*I, *Pac*I and *Bci*VI) that have at least one 5′-TpA sequence in their recognition sites [16]. Effusol inhibited the growth of the wheat pathogen fungus *Zymoseptoria tritici* [17]. Juncusol, dehyrojuncuenin B, juncuenin D and jinflexin B, isolated from *J. inflexus* possessed antibacterial activity against MRSA [18].

The antiviral activity of some of the phenanthrenes was also examined. Denbinobin, the most investigated phenanthrene, inhibited human immunodeficiency virus 1 (HIV-1) reactivation induced by tumor necrosis factor α (TNFα), phorbol 12-myristate 13-acetate (PMA) or α-CD3/α-CD28 monoclonal antibodies (mAbs), in a concentration-dependent manner. The compound inhibited the HIV-1-LTR (long terminal repeat) transactivation through modifying the NF-κB pathway, and presumably, its major target is the NF-κB inhibitory (IκBα) protein [19]. Human herpes simplex virus type 2 (HSV-2) mostly causes genital herpes, which is a significant sexually transmitted disease. It can also cause encephalitis in neonates. Acyclovir and penciclovir are used in the management of HSV-2. Valacyclovir and famcyclovir have been approved for the treatment of recurrent genital HSV as well [20]. Drug resistant HSV-2 strains are frequently isolated from immunosuppressed persons; thus, finding alternative drugs would be desirable [21].

In continuation of our work aiming to isolate biologically active compounds from Juncaceae species, *Juncus compressus* Jacq. (*J. compressus*) was investigated. The isolation process was carried out using combined chromatographic methods. The structures were elucidated by 1D, 2D NMR spectroscopic and HR-MS methods. Eleven compounds have been identified, nine phenanthrenes (**1**–**9**), among them two new compounds (Compounds **1** and **2**), and two flavonoids (Compounds **10** and **11**). Compounds **1**–**9** were tested for their antiproliferative and antiviral activities.

## 2. Results and Discussion

### 2.1. Isolation and Structure Determination of the Compounds

**Figure 1:**
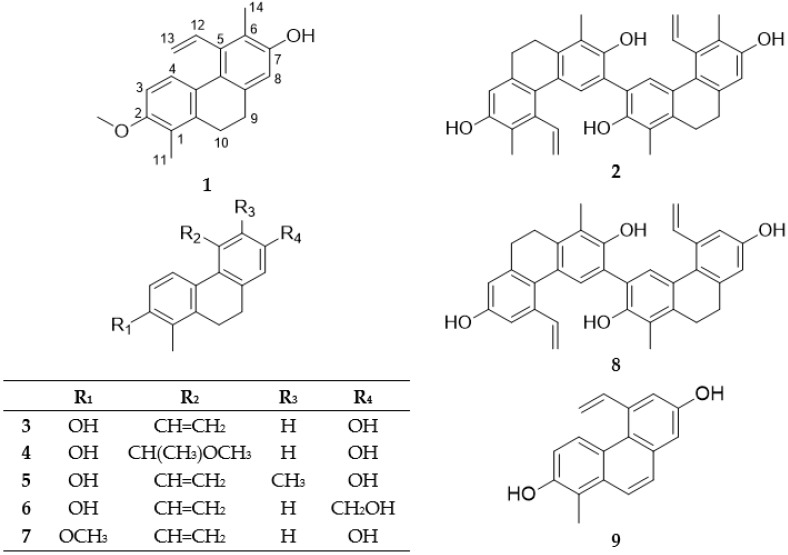
Structures of phenanthrenes (Compounds **1**–**9**) isolated from *J. compressus*.

Dried, whole plant material of *J. compressus* (2.2 kg) was ground and extracted with methanol at room temperature. After concentration, the extract was dissolved in 50% aqueous methanol, and solvent–solvent partition was performed with CH_2_Cl_2_ and EtOAc. The CH_2_Cl_2_ phase was separated and purified with the combination of different chromatographic methods (column chromatography (CC), vacuum liquid chromatography (VLC), gel filtration, rotation planar chromatography (RPC), medium pressure liquid chromatography (MPLC), and high performance liquid chromatography (HPLC) to afford 11 compounds (Figure 1).

The structure determination was carried out by extensive spectroscopic analysis, using one- and two-dimensional NMR (^1^H-^1^H correlation spectroscopy (COSY), heteronuclear single quantum coherence (HSQC), heteronuclear multiple bond correlation (HMBC)) spectroscopy, high-resolution electrospray ionisation mass spectrometry (HRESIMS) and comparison of the spectral data with literature data.

**Figure 2:**
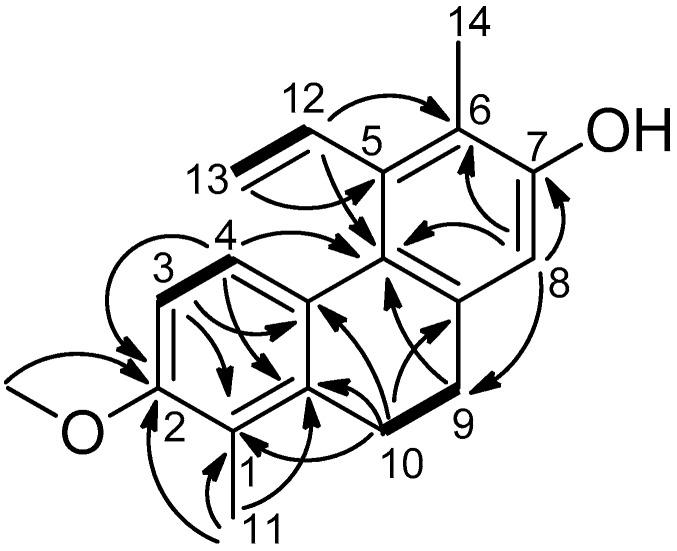
Diagnostic COSY (▬) and HMBC (H→C) correlations of Compound **1**.

Compound **1** was obtained as an amorphous solid. Its HRESIMS provided the molecular formula C_19_H_20_O_2_, through the presence of a peak at *m/z* 281.1532 [M + H]^+^ (calculated for C_19_H_21_O_2_, 281.1573). The ^1^H-NMR spectrum (Table 1) showed signals of two *ortho*-coupled aromatic protons (δ_H_ 6.71 d and 7.62 d), one aromatic proton as a singlet (δ_H_ 6.67), two methyls (δ_H_ 2.29 and 2.22), two methylenes (δ_H_ 2.64 and 2.70), a vinylic system at δ_H_ 6.78, 5.52, and 5.25 (C-12, C-13), and a signal of a methoxy group (δ_H_ 3.85). In the ^1^H NMR spectrum, two methylene signals (H_2_-9, H_2_-10) indicated this compound to be a 9,10-dihydrophenanthrene derivative. In the J-modulated spin-echo (JMOD) spectrum, the presence of 19 carbon signals was detected (Table 1). In the ^1^H-^1^H COSY spectrum, correlations were observed between δ_H_ 6.71 d and δ_H_ 7.62 d (H-3–H-4), δ_H_ 2.64 m and δ_H_ 2.70 m (H-9–H-10), and δ_H_ 6.78 dd and δ_H_ 5.52 d, 5.25 d (H-12–H-13). The methoxy group was placed to C-2 as confirmed by HMBC correlations of the OCH_3_ (δ_H_ 3.85) with C-2 (δ_C_ 156.2) (Figure 2). The location of the methyl groups was also concluded from the HMBC spectrum, as a proton signal at δ_H_ 2.22 (H_3_-11) showed correlations with δ_C_ 122.7 (C-1), 139.5 (C-1a), and 156.2 (C-2), whereas the signal at δ_H_ 2.29 (H_3_-14) was found to be in correlation with δ_C_ 120.6 (C-6), 137.1 (C-5) and 152.5 (C-7). The position of the vinyl group was verified according to the HMBC correlation between δ_H_ 5.52 d, and 5.25 d and δ_C_ 137.1 (CH_2_-13–C-5).

The NOESY correlations further confirmed the structure of Compound **1**. Overhauser effects were detected between H-3/H-4, H-3/OCH_3_-2, OCH_3_-2/H_3_-11, H-8/H-9, and H_3_-14/H-12.

All of the above evidence confirmed the planar structure of Compound **1** named as compressin A (Figure 1).

Compound **2** was obtained as an amorphous solid. In the ^1^H-NMR spectrum (Table 2), signals of two methyls (δ_H_ 2.28 and 2.27), two singlets of aromatic protons (δ_H_ 7.66 and 6.66 s), a vinyl group (δ_H_ 6.77 dd/δ_H_ 5.57 d and 5.25 d), and two hydroxy groups (δ_H_ 5.56 s and 4.73 s) were observed (Table 2). The presence of protons at δ_H_ 2.67 m and 2.62 m (2 × 2H) indicated this compound to be a dihydrophenanthrene according to the saturated C-9–C-10 bond in the B ring. Its JMOD spectrum (Table 2) showed 18 signals close similar to juncusol [22], and the HRESIMS established a molecular formula C_36_H_34_O_4_ (*m/z* 531.4064 [M + H]^+^, calcd. for C_36_H_35_O_4_, 531.4607), suggesting the dimeric nature of Compound **2**. Moreover, the NMR data of Compound **2** were found to be greatly similar to those of juncusol, except of the replacement of a methine group at C-3 of juncusol by a quaternary carbon (δ_C_ 116.4, C-3) in Compound **2**.

**Figure 3:**
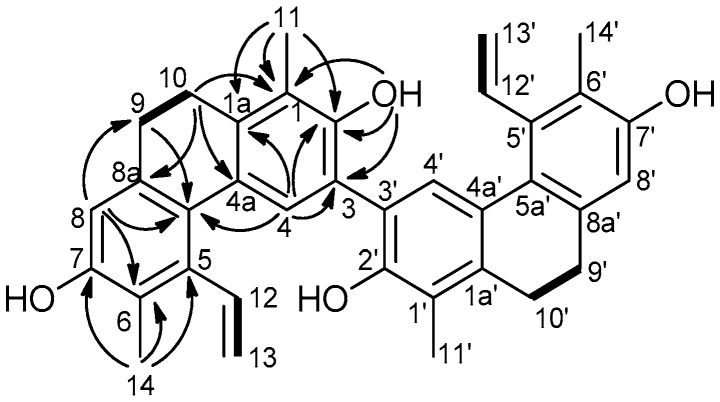
Diagnostic COSY (▬) and HMBC (H→C) correlations of Compound **2**.

In the ^1^H-^1^H COSY spectrum, two correlations were observed between δ_H_ 6.77 dd and 5.57 dd, and 5.25 dd (CH_2_-13–CH-12, CH_2_-13′–CH-12′), and between δ_H_ 2.67 m and 2.62 m (H-9–H-10, H-9′–H-10′) (Figure 3). The vinyl groups according to the HMBC correlation between δ_H_ 5.25 (H-13,13′) and δ_C_ 137.8 are connected to C-5 and C-5′. The position of the methyl groups were also indicated by the HMBC spectrum, where δ_H_ 2.28 (H-11,11′) was correlated with quaternary carbon atoms δ_C_ 122.0 (C-1,1′), 138.6 (C-1a,1a′) and 147.6 (C-2,2′), and protons at δ_H_ 2.27 s (H_3_-14) were in correlation with δ_C_ 120.7 (C-6,6′), 137.8 (C-5,5′) and 152.3 (7,7′), respectively (Figure 3). Since Compound **2** showed only 18 carbon signals instead of 36 in the JMOD spectrum, it should be a symmetric dimer. From the above findings, Compound **2** was thus proposed to be a dimer composed of two juncosol units. The linkage between the monomeric phenanthrene units based on the HMBC correlations detected between δ_H_ 7.66 (H-4,4′) and δ_C_ 116.4 (C-3,3′) and 147.6 (C-2,2′) was determined to be 3-3′.

**Figure 4:**
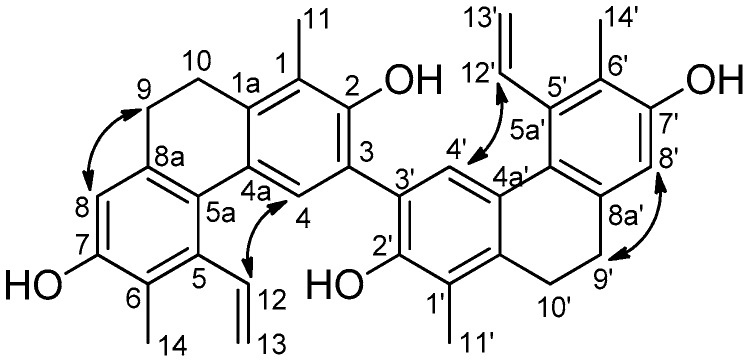
NOESY correlations of Compound **2**.

The NOESY correlations confirmed the structure of Compound **2**. Overhauser effects were detected between H-4,4′/H-12,12′, H-8,8′/H-9,9′, and H_3_-14,14′/H-13,13′ (Figure 4). On the basis of the above findings, the structure of this compound was established as depicted in structural formula 2, and named as compressin B (Figure 1).

Besides the new compounds, compressin A (Compound **1**) and compressin B (Compound **2**), five dihydrophenanthrenes (effusol (Compound **3**), effususol (Compound **4**), juncusol (Compound **5**), 2-hydroxy-1-methyl-7-oxymethylene-5-vinyl-9,10-dihydrophenanthrene (Compound **6**), 7-hydroxy-1-methyl-2-methoxy-5-vinyl-9,10-dihidrophenanthrene (Compound **7**)), one phenanthrene dimer (effususin A (Compound **8**)), one phenanthrene (dehydroeffusol (Compound **9**)), and two flavonoids (apigenin (Compound **10**) and luteolin (Compound **11**)) were also isolated from *J. compressus*. Their structures were determined by analysis of MS, 1D and 2D NMR spectra, and by comparison with literature data [10,22,23,24,25,26,27].

All compounds were isolated for the first time from *J. compressus*. It can be observed that the connection of a methyl group at C-1, methoxy-, or hydroxy-substituents on C-2, and vinyl group on C-5 are characteristic features of the isolated phenanthrenes. Effusol (Compound **3**) can be described as the main compound of this plant with regard to the isolation yield. Juncusol (Compound **5**) is a generally occurring phenanthrene as it was isolated from all investigated Juncaceae species. Compound **7** was isolated previously from *J. acutus* and *J. subulatus*, effusol (Compound **3**) from *J. acutus*, *J. effusus*, *J. maritimus*, *J. setchuensis* and *J. subulatus*, effususol (Compound **4**) from *J. effusus*, Compound **6** from *J. acutus*, *J. effusus*, and *J. subulatus*, effususin A (Compound **8**) from *J. effusus*, and dehydroeffusol (Compound **9**) from *J. acutus*, *J. effusus* and *J. setchuensis* [8], respectively.

### 2.2. Antiproliferative Activity of the Isolated Phenanthrenes

The isolated phenanthrenes (Compounds **1**–**9**) were tested for their antiproliferative activity against three human tumor cell lines (HeLa and SiHa (cervix adenocarcinoma) and A2780 (ovarian carcinoma)) using the 3-(4,5-dimethylthiazol-2-yl)-2,5-diphenyltetrazolium bromide (MTT) test with cisplatin as a positive control (Table 3). The HeLa cell line proved to be the most sensitive with five phenanthrenes (Compounds **2**, **3**, **5**, **6** and **9**) more effective, and two compounds (Compounds **1** and **7**) comparable to the clinically used reference agent cisplatin. Based on the presented results, some structure-activity relationships could be obtained. Since Compound **4** is markedly less effective, a bulky substituent instead of a vinyl group seems to be disadvantageous. Compounds bearing free hydroxy groups (in case of Compound **3**) exerted more pronounced action than its methyl ether (Compound **7**) or its oxymethylene derivative (Compound **6**). The substantial difference in the activities of the isolated dimers (Compounds **2** and **8**) could be attributed to the presence of a methyl group at C-6, which is favored in the monomers, too. Both the other cell lines were substantially less sensitive. Although Compounds **1**, **4** and **8** exhibited some considerable activity against A2780 cells, none of them were comparable to cisplatin. Similarly, none of the presented agents elicited substantial (>40%) inhibition of the cell cancer proliferation during the applied 72 h of incubation against SiHa cells at 10 μM, and therefore, IC_50_ values were not determined.

### 2.3. Antiviral Activity of the Phenanthrenes

First, the cytotoxicity of Compounds **1**–**9** against Vero cells was investigated at concentrations of serial 2-fold dilutions, from 100 μM to 0.78 μM. All compounds were dissolved in DMSO and diluted in culture medium. The maximum concentration of DMSO showed no cytotoxicity for the cells. After 24 h of incubation, cell viability was determined using MTT test. The compounds showed a CC_50_ value higher than 100 µM (data not shown).

To evaluate the possible antiviral effect of the phenanthrenes, HSV-2 infected (MOI: 0.01) Vero cells were treated with a series of 2-fold diluted (100–0.78 µM) compounds. Applying the traditional virus yield reduction assay, juncusol (Compound **5**) caused 3.66 log_10_ reduction of HSV-2 yield even at 0.78 µM concentration compared to the titer of the untreated virus control. This result is remarkable because acyclovir, the gold standard of the herpes therapy, produced 1–5.06 log_10_ reduction of HSV-2 yield at 6.25 µM concentration as published earlier [28]. Direct qPCR method was used to validate our aforementioned results to determine the HSV-2 growth inhibition in the cells infected with the virus in the presence of the serial dilutions of phenanthrenes. Similar to the yield reduction assay, inhibition curves based on qPCR results showed that the most potent compound was juncusol (Compound **5**). The maximum HSV-2 growth corresponded to a DNA concentration of Ct ~ 19 (cycles) value as detected by direct qPCR. The compound concentration that decreased the HSV-2 growth and the corresponding DNA content by 50% (IC_50_), increased the qPCR Ct value by approximately one cycle. In addition, the compound concentration that inhibited the HSV-2 growth by 90% (IC_90_), raised the Ct value by ∼3.32 cycles. In the case of Compound **5**, the IC_50_ was ∼25 µM, and IC_90_ was between 25–50 µM (see Supplementary Material Figure S13).

## 3. Materials and Methods

### 3.1. General

Column chromatography (CC) was performed on polyamide (MP Biomedicals Germany GmbH, Hessen, Germany). Normal phase vacuum liquid chromatography (VLC) was carried out on silica gel (Kieselgel 60 GF_254_,15 µm, Merck, Darmstadt, Germany). Medium pressure liquid chromatography (MPLC) was processed with a Combi Flash Rf^+^ Lumen instrument (Teledyne Isco, Lincoln, NE, USA). Rotation planar chromatography (RPC) was carried out by a Chromatotron instrument (Model 8924, Harrison Research, T-Squared Technology, Inc., San Bruno, CA, USA). Sephadex LH-20 (25–100 μm, Pharmacia Fine Chemicals, Piscataway, NJ, USA) was used for gel filtration. The HPLC system was comprised of a Waters 600 controller, Waters 600 pump, and Waters 2998 photodiode array detector. The data were acquired and processed with the Empower software (Empower 2, Waters Corporation, Milford, MA, USA).

NMR spectra were recorded in CD_3_OD and CDCl_3_ on a BrukerAvance DRX 500 spectrometer at 500 MHz (^1^H) and 125 MHz (^13^C). The signals of the deuterated solvents were taken as references. The chemical shift values (δ) were given in ppm and coupling constants are in Hz. Two-dimensional (2D) experiments were performed with a standard Bruker software. In the ^1^H-^1^H COSY, HSQC and HMBC experiments, gradient-enhanced versions were applied. The high resolution MS spectra were acquired on a Thermo Scientific Q-Exactive Plus Orbitrap mass spectrometer equipped with an electrospray ionization electrospray ionization (ESI) ion source in positive ionization mode. The resolution was over 1 ppm. The data were acquired and processed with the MassLynx software. All solvents used for CC were of at least analytical grade (VWR Ltd., Szeged, Hungary).

### 3.2. Plant Material

The whole plant of *Juncus compressus* were collected in June 2014, near Gyula (GPS coordinates are 46°35′46.19″ N, 21°10′18.97″ E). The corresponding voucher specimen (No. 876) has been deposited at the Herbarium of the Department of Pharmacognosy, University of Szeged.

### 3.3. Extraction and Isolation

The air-dried, whole plant of *J. compressus* (2.2 kg) was ground and percolated with methanol (150 L) at room temperature. The crude methanol extract was concentrated under reduced pressure (323.7 g). The residue was dissolved in 50% methanol and subjected to solvent–solvent partitioning with dichloromethane (CH_2_Cl_2_) (6 L) and ethyl acetate (EtOAc) (3 L), respectively. After evaporation, the dichloromethane fraction (96.4 g) was chromatographed on a polyamide column with mixtures of methanol and water (1:1, 4:1 (10 L and 32 L, respectively); each eluent was collected as a fraction). The fraction obtained from the polyamide column with MeOH-H_2_O 4:1 (27.5 g) was further chromatographed by VLC on silica gel with a gradient system of cyclohexane-EtOAc-MeOH (from 98:2:0 to 5:5:1 (1000 mL/eluent); volume of each fraction was 100 mL) to yield 28 major fractions (I–XXVIII).

Fraction IX (1.3 g) was separated by VLC on silica gel with *n*-hexane-diethyl ether solvent system (from 1:0 to 0:1) to obtain five sub-fractions (the volume of the collected fractions was 25 mL). Sub-fraction IX/4 was subjected to Sephadex LH-20 column chromatography eluting with CHCl_3_-MeOH 1:1 to yield Compound **1** (5.7 mg). Fraction X (1.3 g) was separated by MPLC, using *n*-hexane-EtOAc gradient system (from 1:0 to 0:1), to yield 13 sub-fractions (the volume of the collected fractions was 25 mL). Sub-fraction X/6 was purified by gel filtration, applying CHCl_3_-MeOH 1:1 as eluent to yield Compound **2** (3.9 mg). Fraction XII (389 mg) was further chromatographed by RPC on silica gel using *n-*hexane-acetone-MeOH gradient system (from 8:2:0 to 0:0:1; volume of the collected fractions was 15 mL) to obtain 4 sub-fractions. Sub-fraction XII/3 was subjected to column chromatography over Sephadex LH-20 gel, eluting with CHCl_3_-MeOH 1:1. Sub-fraction XII/3/a was purified by NP-HPLC with a Zorbax Sil (5 µm, 9.4 × 250 mm) column under isocratic conditions, using cyclohexane-EtOAc 8:2 as a mobile phase, at a flow rate of 3.5 mL/min to yield Compound **7** (*t*_R_ = 6.0 min, 9.7 mg). Fraction XIII (2 g) was rechromatographed on silica gel, applying *n*-hexane-EtOAc-MeOH 95:5:0–1:1:1 as mobile phase to get 6 sub-fractions (the volume of the collected fractions was 15 mL). Sub-fraction XIII/2 was further fractionated by Sephadex LH-20 gel chromatography (MeOH-CHCl_3_ 1:1) to obtain three fractions, from which XIII/2/1 resulted in Compound **5** (8.0 mg) after purifying by NP-HPLC (using Zorbax Sil (5 µm, 9.4 × 250 mm) column and cyclohexane-EtOAc 85:15 as an isocratic eluent system at a flow rate of 3 mL/min, *t*_R_ = 8.2 min). Sub-fraction XIII/3 was subjected to column chromatography over Sephadex LH-20 gel (MeOH–CHCl_3_ 1:1) to gain Compound **3** (112 mg). The remaining fraction was purified by RP-HPLC applying a Zorbax octadecylsilane (ODS) (5 µm, 9.4 × 250 mm) column and MeOH-H_2_O isocratic system (7:3, flow rate 3.5 mL/min) and Compound **4** (*t*_R_ = 8.8 min, 4.5 mg) was isolated. Fraction XIII/4 was further separated by gel filtration, using MeOH-CHCl_3_ 1:1 mixture as mobile phase to obtain three sub-fractions. Sub-fraction XIII/4/2 was purified by RP-HPLC using a Zorbax ODS (5 µm, 9.4 × 250 mm) column, and MeOH-H_2_O 6:4 (flow rate 3.5 mL/min) to yield Compound **9** (*t*_R_ = 10.0 min, 4.3 mg). Fraction XV (124 mg) was subjected to column chromatography over Sephadex LH-20 gel, eluting with MeOH-CHCl_3_ 1:1 solvent system, and 3 sub-fractions were obtained, from which XV/2 provided Compound **6** (3.6 mg) after purification by RP-HPLC (Zorbax ODS (5 µm, 9.4 × 250 mm) with isocratic mobile phase (MeOH-H_2_O 85:15); 3.0 mL/min flow rate; *t*_R_ = 4.9 min). Fraction XX (183 mg) was separated by Sephadex LH-20 gel chromatography (MeOH-CHCl_3_ 1:1) to yield three sub-fractions. Sub-fraction XX/3 was further purified by NP-HPLC using a Zorbax Sil (5 µm, 9.4 × 250 mm) column and cyclohexane-EtOAc 85:15 isocratic system, at a flow rate of 3 mL/min to yield Compound **8** (*t*_R_ = 5.5 min, 4.5 mg). Fraction XXVIII (2 g) was further separated by RP-MPLC (MeOH-H_2_O from 0:1 to 1:0) to get nine sub-fractions (volume of collected fractions was 25 mL). Sub-fractions XXVIII/4–7 were further purified by Sephadex LH-20 (CHCl_3_-MeOH 1:1) gel chromatography to yield Compounds **10** (23.3 mg) and 11 (18.5 mg).

#### 3.3.1. Compressin A (Compound **1**)

An amorphous solid; UV (MeOH) *λ*_max_ (log *ε*) 281 (3.66), 217 (3.80) nm; ^1^H and ^13^C-NMR data, see Table 1; HRESIMS *m/z* 281.1532 [M + H]^+^ (calculated for C_19_H_21_O_2_, 281.1573).

#### 3.3.2. Compressin B (Compound **2**)

An amorphous solid; ${\left[\alpha \right]}_{D}^{26}$ 0 (*c* 0.1, MeOH); UV (MeOH) *λ*_max_ (log ε) 280 (2.88), 214 (3.24) nm; ^1^H and ^13^C-NMR data, see Table 2; HRESIMS *m/z* 531.4064 [M + H]^+^, calculated for C_36_H_35_O_4_, 531.4607.

### 3.4. Antiproliferative Assay

The antiproliferative properties of the isolated phenanthrenes were determined on a panel of human malignant cell lines isolated from cervical (HeLa and SiHa) and ovarian cancers (A2780) purchased from the European Collection of Cell Cultures (ECCAC, Salisbury, UK). Cells were cultivated in minimal essential medium supplemented with 10% fetal bovine serum, 1% non-essential amino acids and an antibiotic-antimycotic mixture. All media and supplements were obtained from Lonza Group Ltd., (Basel, Switzerland). Near-confluent cancer cells were seeded onto a 96-well microplate (5000 cells/well) and after an overnight standing, medium containing the tested compounds at 10 and 30 µM was added. After incubation for 72 h under cell culture conditions, the living cells were assayed by the addition of 20 µL of 5 mg/mL 3-(4,5-dimethylthiazol-2-yl)-2,5-diphenyltetrazolium bromide (MTT) solution. MTT was converted by intact mitochondrial enzymes and precipitated as purple crystals during a 4 h contact period. The medium was then removed and the formazan was dissolved in 100 µL of DMSO during a 60 min period of shaking at 37 °C. Finally, the reduced MTT was assayed at 545 nm using a microplate reader [29,30]. In the case of the most active compounds (i.e., higher than 40% growth inhibition at 10 µM), the assays were repeated with a set of dilutions, sigmoidal concentration-response curves were fitted to the determined data and the IC_50_ values were calculated by means of GraphPad Prism 5.01 (GraphPad Software, San Diego, CA, USA). All in vitro experiments were carried out on two microplates with five parallel wells. Stock solutions of the tested compounds (10 mM) were prepared in DMSO. The highest DMSO content of the medium (0.3%) did not have any substantial effect on cell proliferation. Cisplatin (Ebewe Pharma GmbH, Unterach, Austria) was used as a reference agent.

### 3.5. Anti-HSV-2 Assay

#### 3.5.1. Cultivation and Quantification of Herpes Viruses

The HSV-2 strain was (donated by Dr. Ilona Mucsi, University of Szeged, Szeged, Hungary) grown in Vero cells (ATCC, Wesel, Germany) and the infectivity was measured in the same cell line by using the plaque titration method.

#### 3.5.2. MTT Assay for Determination of Non-Toxic Concentration of Compounds in Vero Cells

An MTT assay was carried out to identify the highest non-toxic concentration of Compounds **1**–**9** with potential antiviral activity. The medium was removed from Vero cells after an overnight period of growing, and fresh medium complemented with serial 2-fold dilutions of all compounds was added to three parallel wells for each concentration. After 24 h, the MTT assay was performed as described earlier [31].

#### 3.5.3. Assay for Testing Antiviral Activity on Vero Cells

The antiviral activity of phenanthrenes was investigated in Vero cells. Cells were seeded in 96-well plates and were infected with HSV-2 at a multiplicity of infection (MOI) of 0.01. After a 1 h adsorption period, the inoculum was removed, the cultures were washed twice, and culture medium containing the plant compounds in different concentrations was added. After a 24 h incubation period, the cultures were washed with phosphate buffered saline, and finally 100 μL Milli-Q water (MQ) (Millipore, Billerica, MA, USA) was added to the cells, and the cultures in the plate were frozen.

#### 3.5.4. Antiviral Activity Using qPCR

DNA release from the infected host cells was achieved by two freeze-thaw cycles, and 1 μL of the lysates were used directly in a qPCR. Each antiviral test was performed in three parallel wells. The qPCR was performed using the Bio-Rad CFX96 real time system, as described earlier [32]. Briefly, the HSV-2 gD2 gene specific primer pair was applied during the qPCR process. The primer sequences were the following: gD2: 5′-TCA GCG AGG ATA ACC TGG GA-3′, 5′-GGG AGA GCG TAC TTG CAG GA-3′. The qPCR mixture consisted of 5 μL SsoFast™ EvaGreen^®^ Supermix (Bio-Rad, Hercules, CA, USA), 1–1 μL forward and reverse primers (10 pmol/µL each), 1 μL template and 2 μL MQ water was added to get a final volume of 10 μL. After a 10 min polymerase activation step at 95 °C, 40 PCR cycles of 20 s at 95 °C and 1 min at 69 °C were performed. Fluorescence intensity was detected at the end of the annealing-extension step. The specificity of amplification was confirmed by melting curve analysis. For each PCR, the cycle threshold (Ct) corresponding to the cycle where the amplification curve crossed the baseline was determined.

#### 3.5.5. Determination of TCID_50_ with Virus Yield Reduction Technique

The virus yield in the supernatants of infected and plant compound-treated cells was determined by the traditional dilution method. Vero cells (6 × 10^4^ cells/well) were seeded onto 96-well flat-bottom plates and cultivated for 24 h at 37 °C at 5% CO_2_ to produce a semi-confluent monolayer. Then the growth medium was removed and 10-fold dilutions of HSV-2 in the absence of the compound (virus control) as well as compound-treated HSV-2-infected cell supernatants were added in quadruplicate and plates were incubated at 37 °C until typical cytopathic effect (CPE) was visible. After 48 h, the CPE of the virus was examined using an inverted microscope, and the titers were estimated according to the Reed-Muench method, expressed as TCID_50_/mL (median tissue culture infective dose). The test compounds’ antiviral activity was measured as the reduction of the viral titer (log_10_) in the presence of each compound, compared to the virus titer of the control sample.

## 4. Conclusions

In the last few decades, numerous novel phenanthrenes (approx. *n* = 90) have been described from six *Juncus* (*J. acutus*, *J. effusus*, *J. inflexus*, *J. roemerianus*, *J. setchuensis*, *J. subulatus*) and one *Luzula* (*L. luzuloides*) species. Our work resulted in the identification of nine (Compounds **1**–**9**) phenanthrenes (among them two dimers), substituted with hydroxy, methyl, methoxy, oxymethylene, and vinyl groups. Two components (Compounds **1** and **2**) are novel natural compounds. The other phenanthrenes (Compounds **3**–**9**) were isolated previously only from other Juncaceae species. All compounds were isolated from *J. compressus* for the first time. Some of the isolated phenanthrenes and one of the dimers elicited remarkable antiproliferative actions against human cancer cells. Juncusol (Compound **5**) was proved to be active against HSV-2. Our results confirmed that phenanthrenes are promising molecules in the search for new antiproliferative agents.

## Figures and Tables

**Table 1:** NMR spectroscopic data for compressin A (Compound **1**) (500 MHz (^1^H), 125 MHz (^13^C), δ in ppm, CDCl_3_).

Position	δ_H_ (*J* in Hz)	δ_C_, Type	HMBC (H→C)
1		122.7, C	
1a		139.5, C	
2		156.2, C	
3	6.71, d (8.6)	106.9, CH	1, 2, 4a
4	7.62, d (8.6)	128.3, CH	1a, 2, 5a
4a		127.5, C	
5		137.1, C	
5a		127.6, C	
6		120.6, C	
7		152.5, C	
8	6.67, s	113.2, CH	5a, 6, 7, 9
8a		137.9, C	
9	2.64, m (2H)	30.5, CH_2_	1a, 5a, 8, 8a
10	2.70, m (2H)	25.9, CH_2_	1, 1a, 4a, 8a
11	2.22, s	11.9, CH_3_	1, 1a, 2
12	6.78, dd (17.9, 11.4)	137.8, CH	5, 5a, 6
13	5.52, d (11.4) 5.25, d (18.0)	119.8, CH_2_	
14	2.29, s	13.4, CH_3_	5, 6, 7
OCH_3_	3.85, s	55.7, CH_3_	2

**Table 2:** NMR spectroscopic data for compressin B (Compound **2**) (500 MHz (^1^H), 125 MHz (^13^C), δ in ppm, CDCl_3_).

Position	δ_H_ (*J* in Hz)	δ_C_, Type	HMBC (H→C)
1, 1′		122.0, C	
1a, 1a′		138.6, C	
2, 2′		147.6, C	
3, 3′		116.4, C	
4, 4′	7.66, s	127.3, CH	1a, 1a′, 2, 2′, 3, 3′, 5a, 5a′
4a, 4a′		128.1, C	
5, 5′		137.8, C	
5a, 5a′		126.3, C	
6, 6′		120.7, C	
7, 7′		152.3, C	
8, 8′	6.66, s	113.2, CH	5a, 5a′, 6, 6′, 7, 7′, 9, 9′
8a, 8a′		137.2, C	
9, 9′	2.62, m	30.2, CH_2_	1a, 1a′, 5a 5a′, 8, 8′, 8a, 8a′, 10, 10′
10, 10′	2.67, m	25.6, CH_2_	1, 1′, 1a, 1a′, 4a, 4a′, 8a, 8a′, 9, 9′
11, 11′	2.28, s	12.6, CH_3_	1, 1′, 1a, 1a′, 2, 2′
12, 12′	6.77, dd (18.0, 11.4)	137.1, CH	13, 13′
13, 13′	5.57, dd (11.4, 1.7) 5.25, dd (18.0, 1.7)	120.5, CH_2_	12, 12′
14, 14′	2.27, s	13.3, CH_3_	5, 5′, 6, 6′, 7, 7′
2-OH, 2′-OH	5.56, s		1, 1′, 2, 2′, 3, 3′
7-OH, 7-OH’	4.73, s		

**Table 3:** Antiproliferative effects of the isolated phenanthrenes (**1**–**9**) on human cancer cell lines.

Compound	Concentration (μM)	Growth Inhibition (%) ± SEM (Calculated IC_50_ Value (μM))		
		HeLa	SiHa	A2780
**1**	10	41.67 ± 1.10	– *	47.64 ± 1.58
	30	93.73 ± 0.36	38.56 ± 0.78	73.00 ± 0.47
		11.27		13.19
**2**	10	92.03 ± 0.44	–	–
	30	92.38 ± 0.15	32.82 ± 0.88	64.85 ± 1.95
		1.86		
**3**	10	96.37 ± 0.28	–	–
	30	97.77 ± 0.27	–	50.53 ± 1.59
		3.68		
**4**	10	–	–	18.33 ± 0.42
	30	30.45 ± 1.91	14.23 ± 2.75	72.05 ± 1.06
**5**	10	97.71 ± 0.37	17.05 ± 2.37	18.06 ± 1.26
	30	97.76 ± 0.23	29.26 ± 2.00	63.43 ± 0.98
		1.31		
**6**	10	96.80 ± 0.28	–	–
	30	98.77 ± 0.24	10.25 ± 1.47	–
		4.17		
**7**	10	45.38 ± 1.57	14.23 ± 0.98	–
	30	92.36 ± 0.23	29.48 ± 3.36	63.24 ± 2.54
		10.66		
**8**	10	–	–	–
	30	10.45 ± 0.47	14.42 ± 1.48	72.73 ± 1.82
**9**	10	75.15 ± 2.48	–	–
	30	96.50 ± 0.32	28.69 ± 2.01	57.27 ± 2.58
		7.75		
Cisplatin	10	42.61 ± 2.33	88.64 ± 0.50	83.57 ± 1.21
	30	99.93 ± 0.26	90.18 ± 7.78	95.02 ± 0.28
		12.43	7.84	1.30

* Growth inhibition values less than 10% were considered insignificant and the exact results are not given for simplicity.

## Supplementary Materials

Supplementary materials are available online. Figure S1–S12: NMR spectra of Compounds **1** and **2**, Figure S13: Antiviral effect of Compounds **1**–**9**.
