# Supplementary material for: Phenanthrenes from Juncus Compressus Jacq. with Promising Antiproliferative and Anti-HSV-2 Activities
Source: Molecules. 2018 Aug 20;23(8):2085. doi: 10.3390/molecules23082085 (PMC6222307; doi:10.3390/molecules23082085)
Supplement: Supplementary file 1 [file molecules-23-02085-s001.pdf]

Supporting Information

Phenanthrenes from *Juncus compressus* Jacq. with promising antiproliferative and anti-HSV-2 activities

Csaba Bús [1], Norbert Kúsz [1], Gusztáv Jakab [2], Seyyed Ashkan Senobar Tahaei [3], István Zupkó [3,4], Valéria Endrész [5], Anita Bogdanov [5], Katalin Burián [5], Boglárka Csupor-Löffler [1], Judit Hohmann [1,4], Andrea Vasas[1]*

[1] *Department of Pharmacognosy, University of Szeged, 6720 Szeged, Hungary*

[2] *Institute of Environmental Sciences, Faculty of Water and Environmental Management, Szent István University Szarvas, H-5540 Szarvas, Hungary*

[3] *Department of Pharmacodynamics and Biopharmacy, University of Szeged, 6720 Szeged, Hungary*

[4] *Interdisciplinary Centre of Natural Products, University of Szeged, 6720 Szeged, Hungary*

[5] *Department of Medical Microbiology and Immunobiology, University of Szeged, 6720 Szeged, Hungary*

*Author to whom correspondence should be addressed.

Table of Contents

Figure S1. Page S3
^{1}H-NMR spectrum of compound **1** (500 MHz, $CDCl_3$)

Figure S2. Page S3
JMOD spectrum of compound **1** (125 MHz, $CDCl_3$)

Figure S3. Page S4
HSQC spectrum of compound **1**

Figure S4. Page S4
^{1}H-^{1}H-COSY spectrum of compound **1**

Figure S5. Page S5
HMBC spectrum of compound **1**

Figure S6. Page S5
NOESY spectrum of compound **1**

Figure S7. Page S6
^{1}H-spectrum of compound **2** (500 MHz, $CDCl_3$)

Figure S8. Page S6
JMOD spectrum of compound **2** (125 MHz, $CDCl_3$)

Figure S9. Page S7
HSQC spectrum of compound **2**

Figure S10. Page S7
^{1}H-^{1}H-COSY spectrum of compound **2**

Figure S11. Page S8
HMBC spectrum of compound **2**

Figure S12. Page S8
HMBC spectrum of compound **2**

Figure S13. Page S9
Antiviral effect of compounds **1**–**9**.

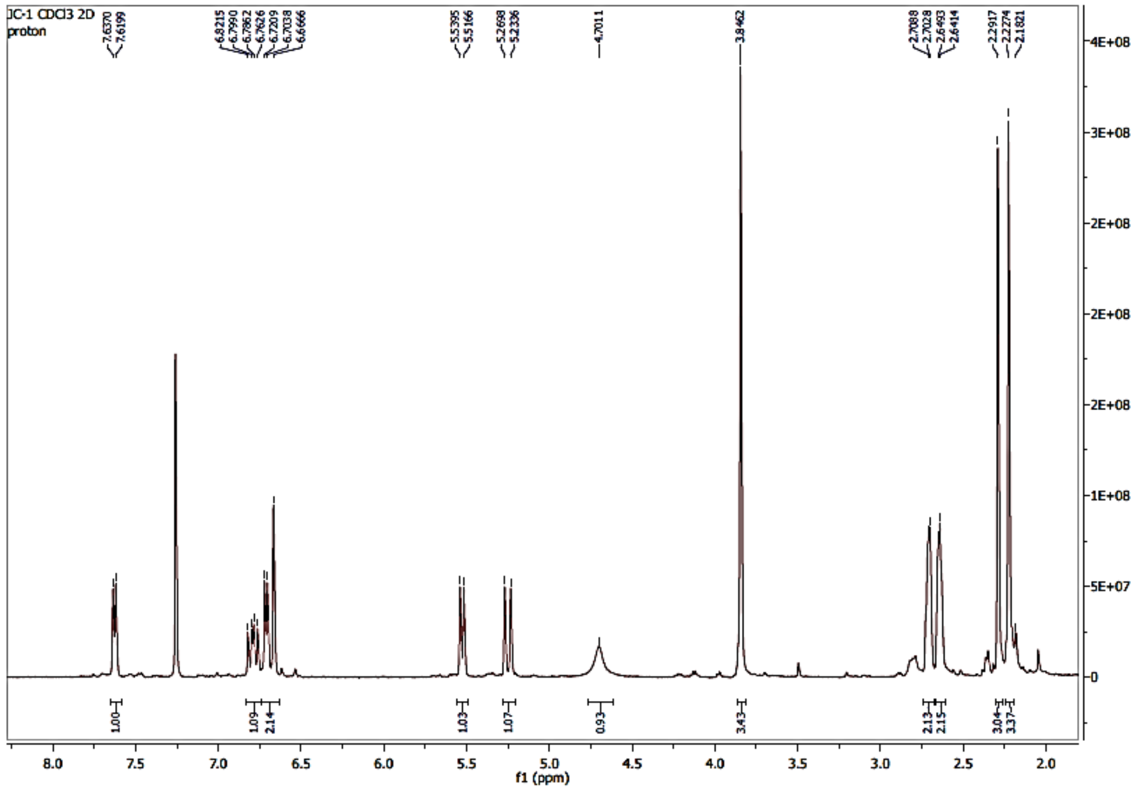

Figure S1. ^{1}H-NMR spectrum of compound **1** (500 MHz, $CDCl_3$)

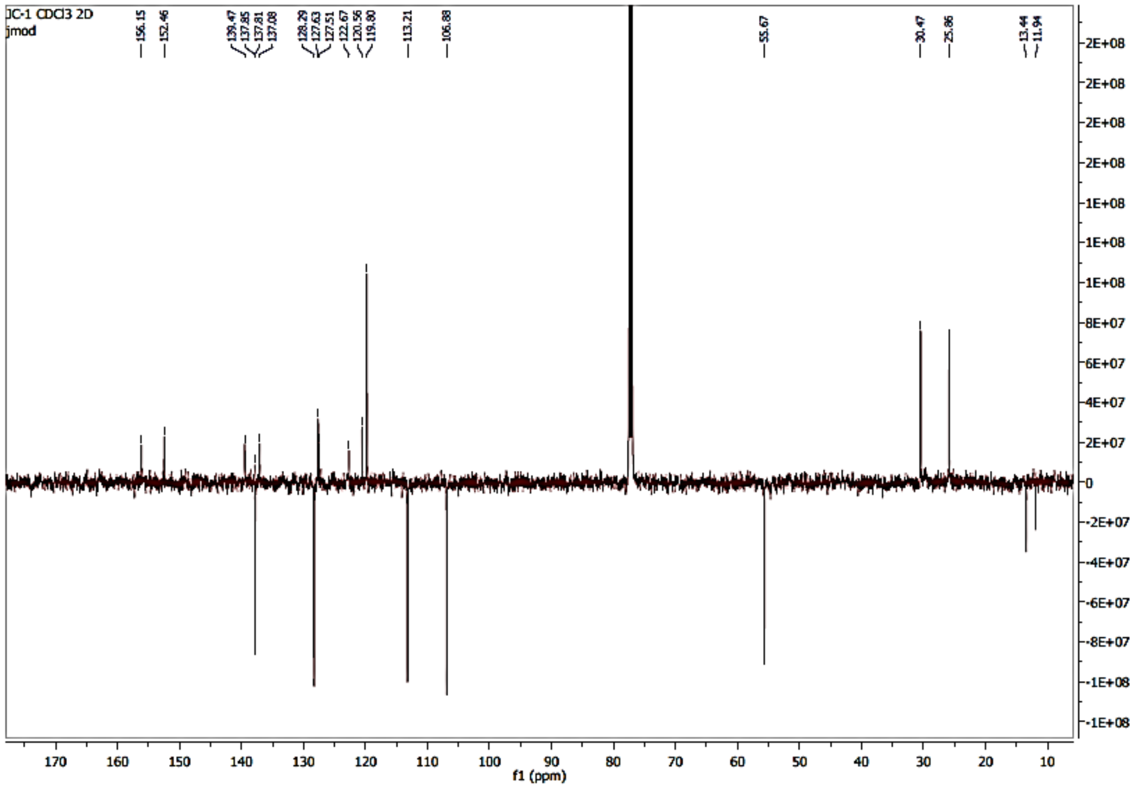

Figure S2. JMOD spectrum of compound **1** (125 MHz, $CDCl_3$)

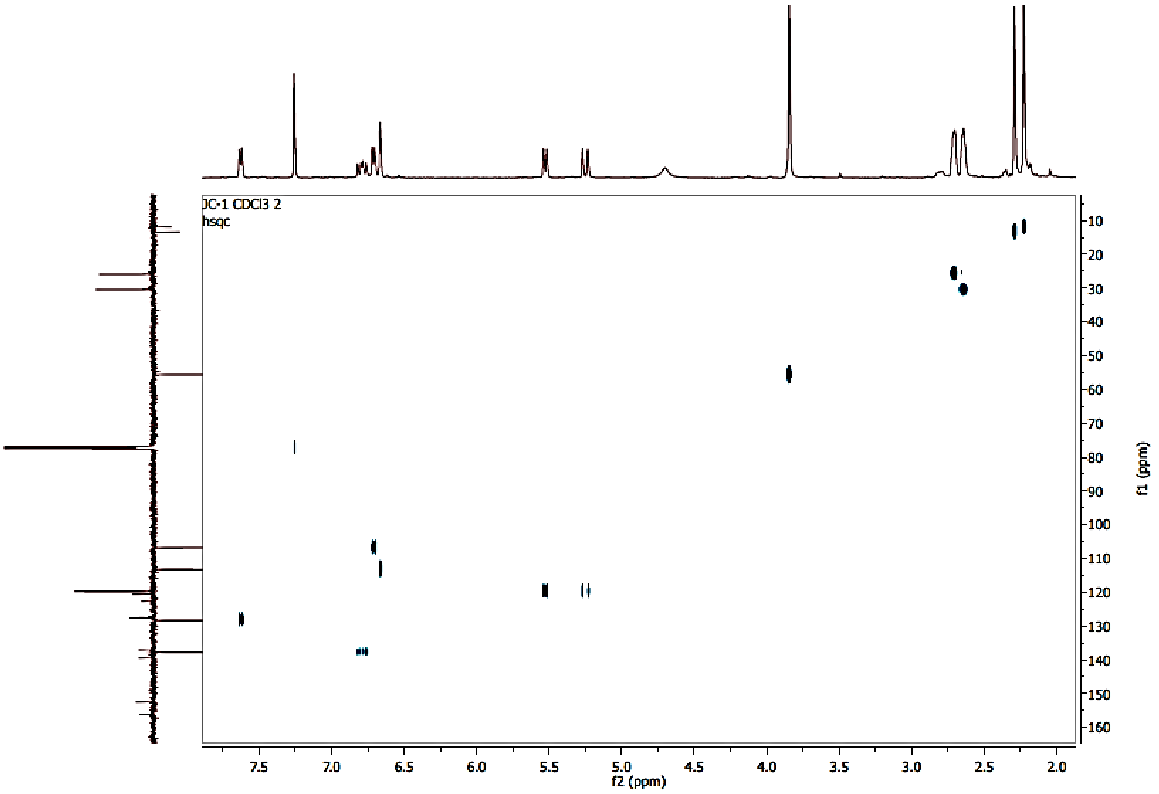

Figure S3. HSQC spectrum of compound **1**

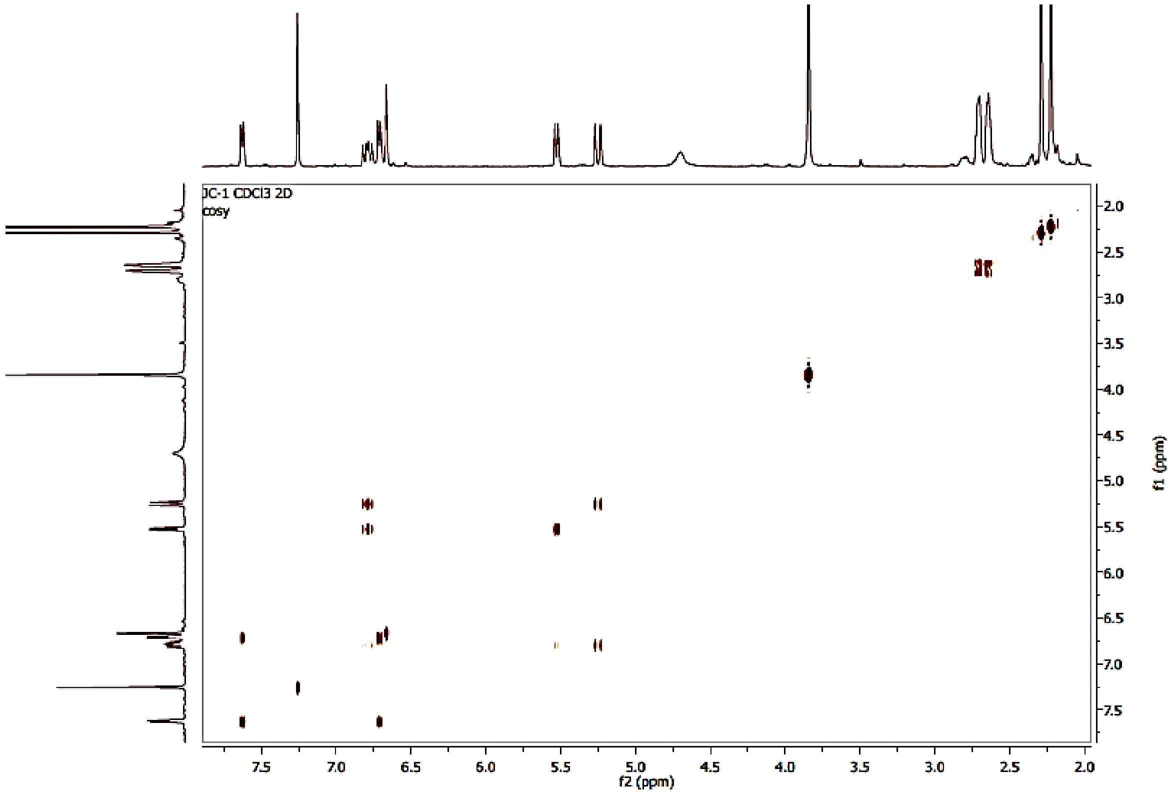

Figure S4. ^{1}H-^{1}H-COSY spectrum of compound **1**

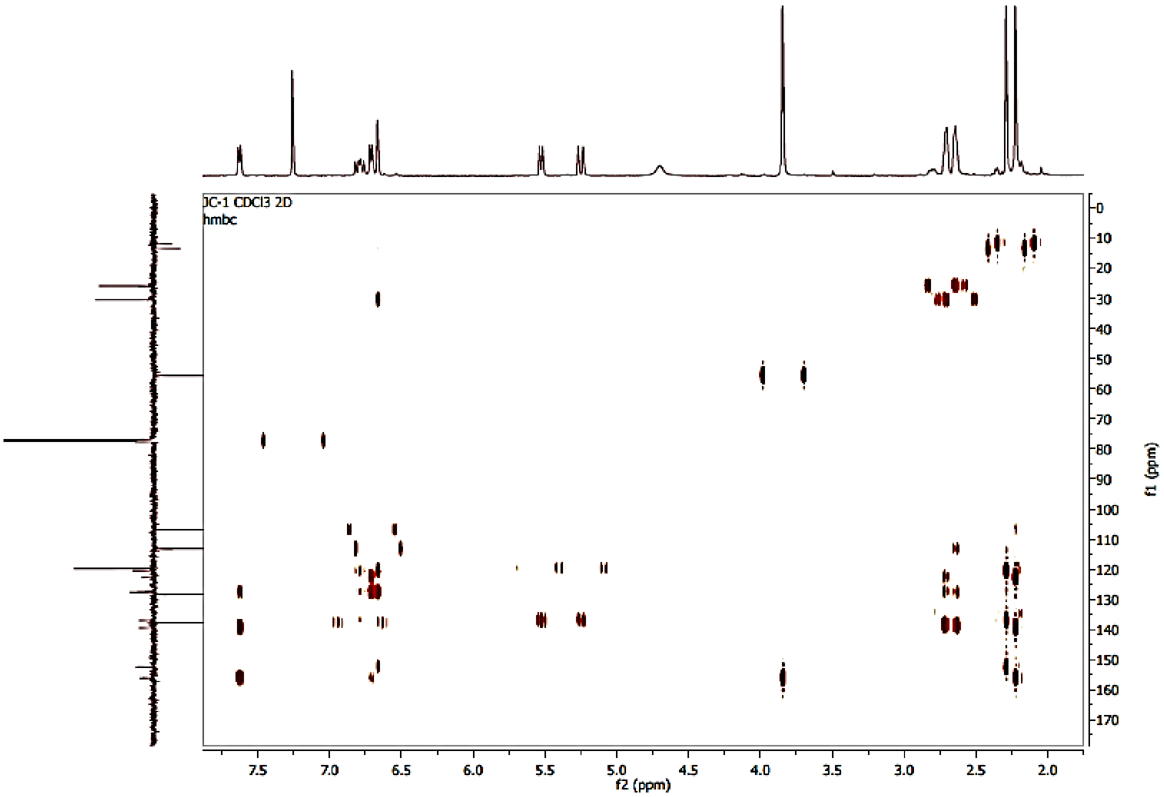

Figure S5. HMBC spectrum of compound **1**

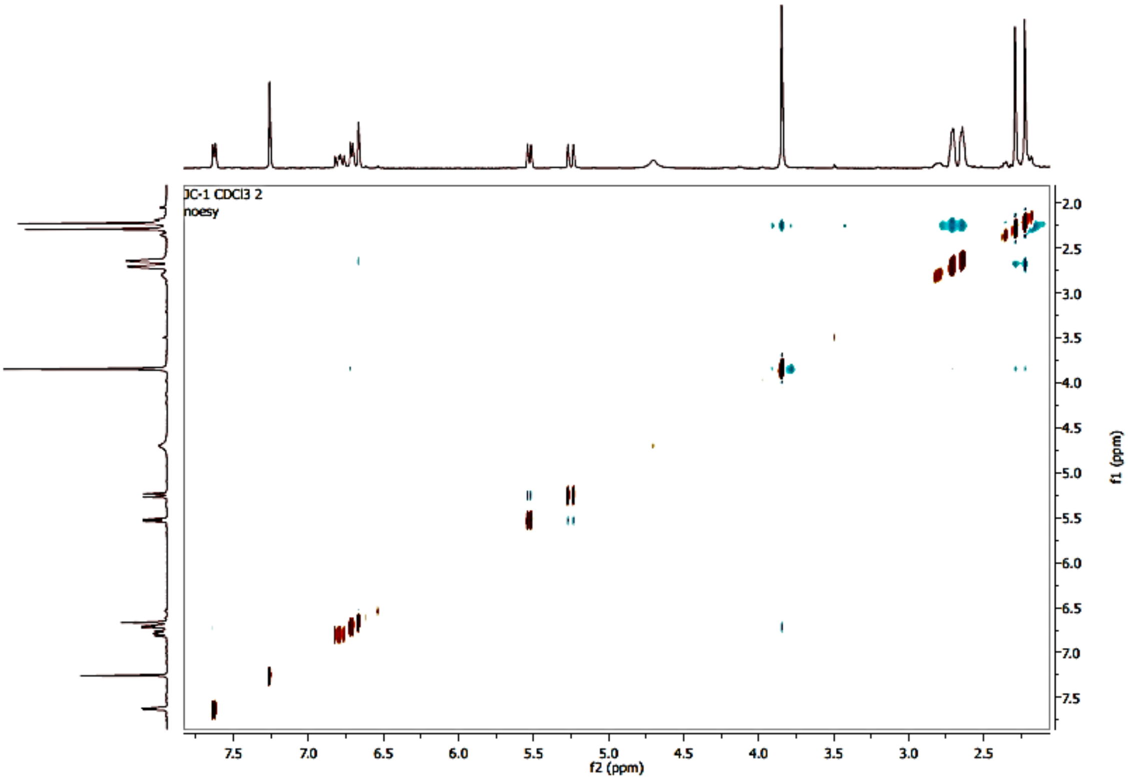

Figure S6. NOESY spectrum of compound **1**

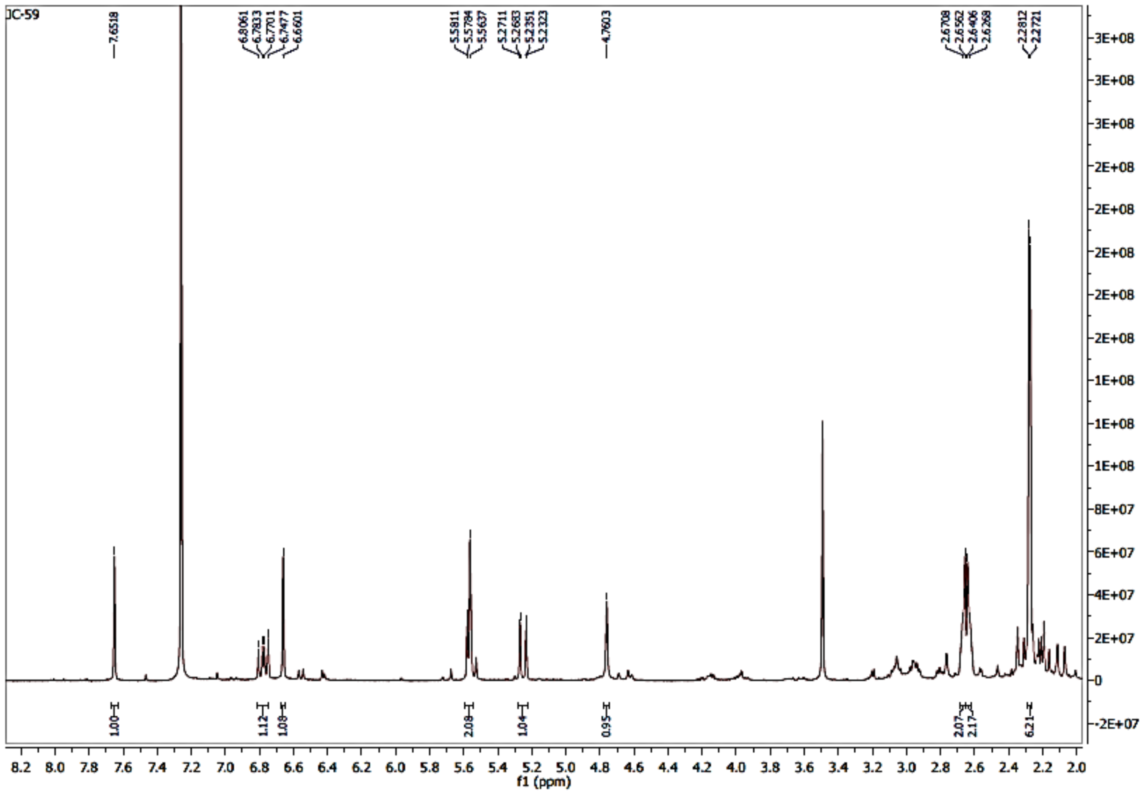

Figure S7. ^{1}H-spectrum of compound **2** (500 MHz, $CDCl_3$)

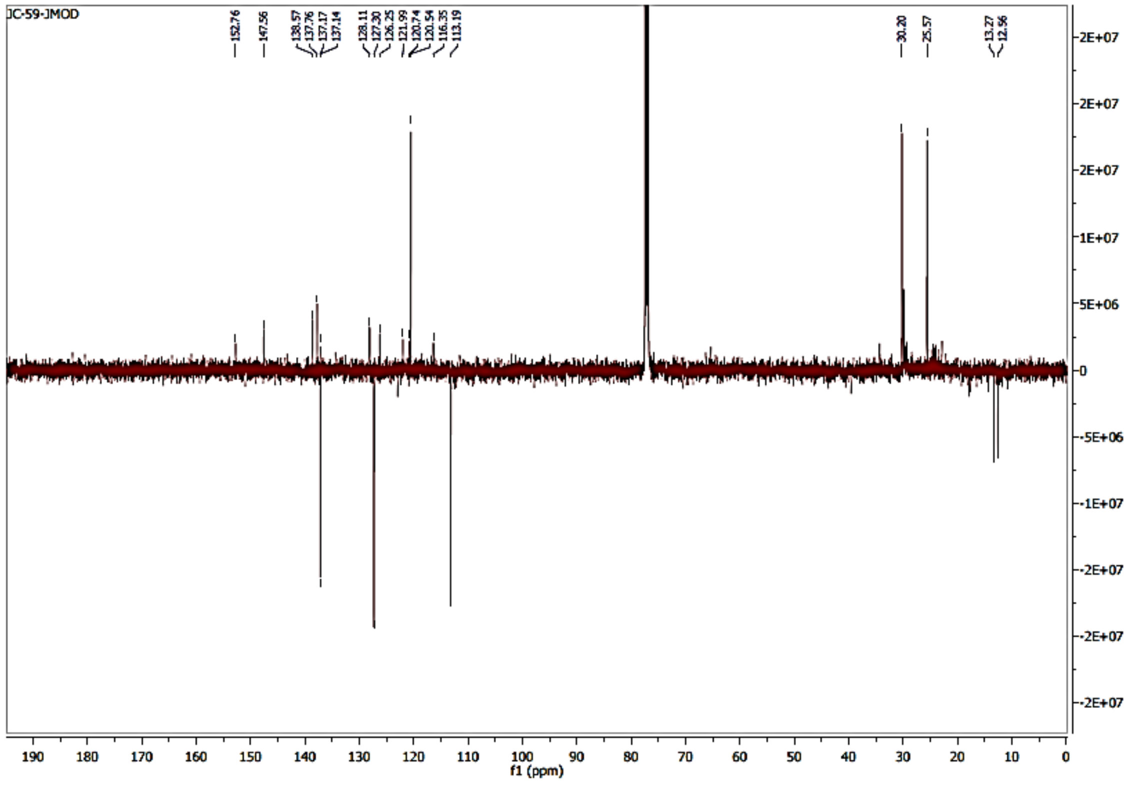

Figure S8. JMOD spectrum of compound **2** (125 MHz, $CDCl_3$)

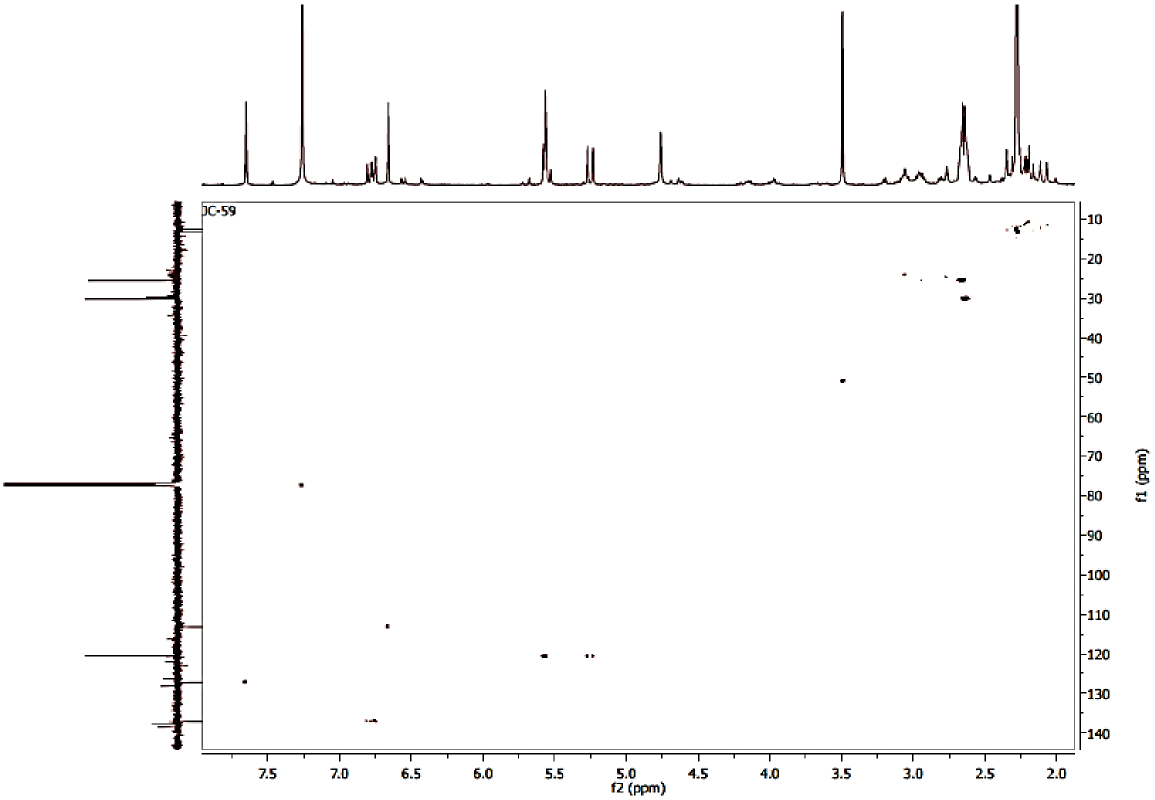

Figure S9. HSQC spectrum of compound **2**

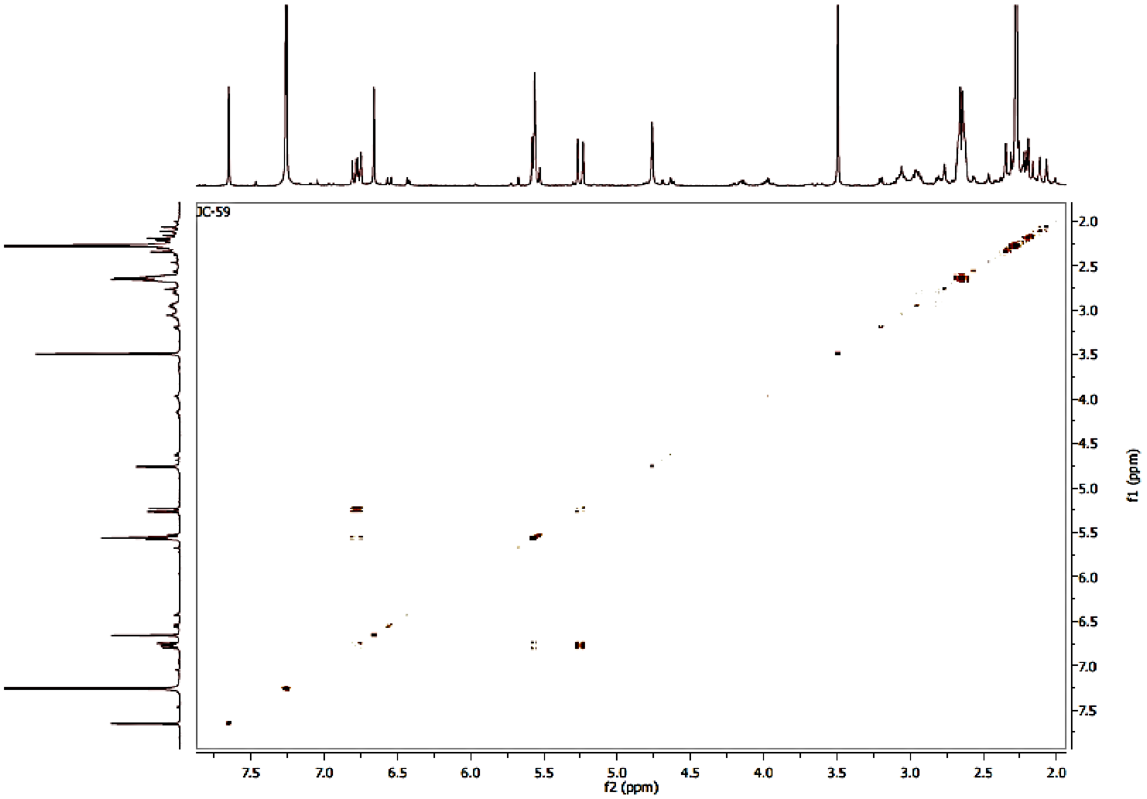

Figure S10. ^{1}H-^{1}H-COSY spectrum of compound **2**

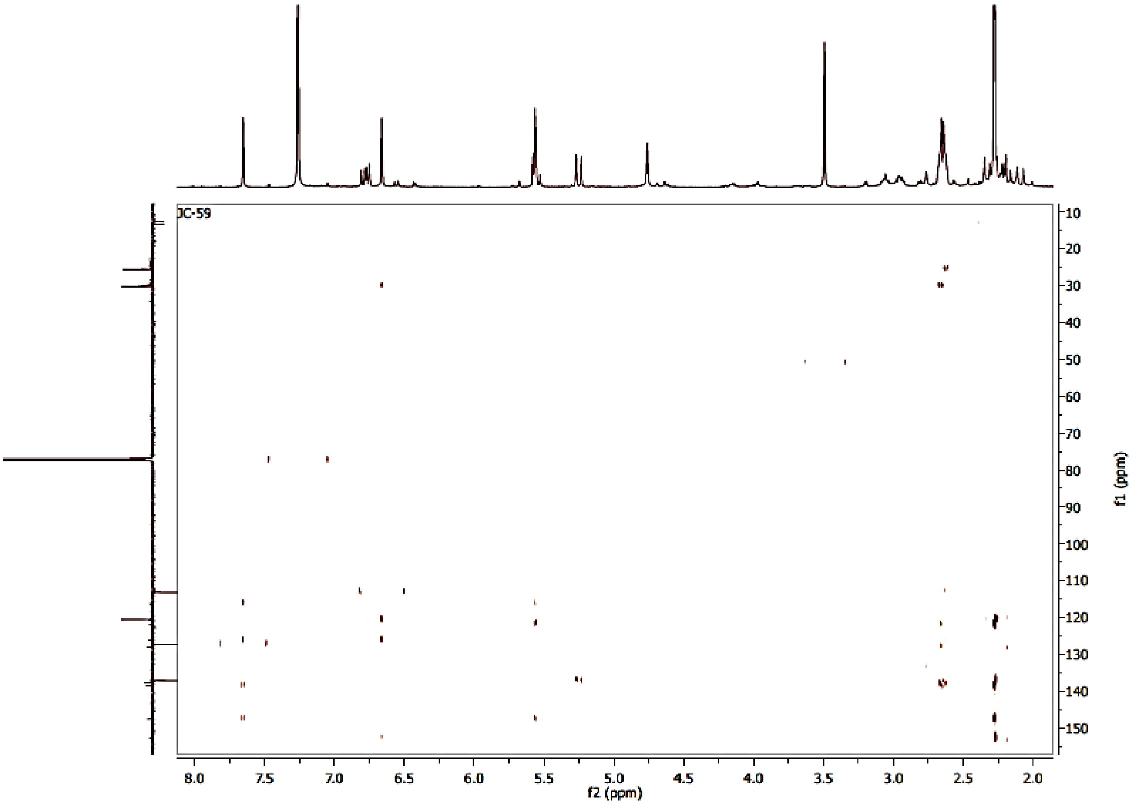

Figure S11. HMBC spectrum of compound **2**

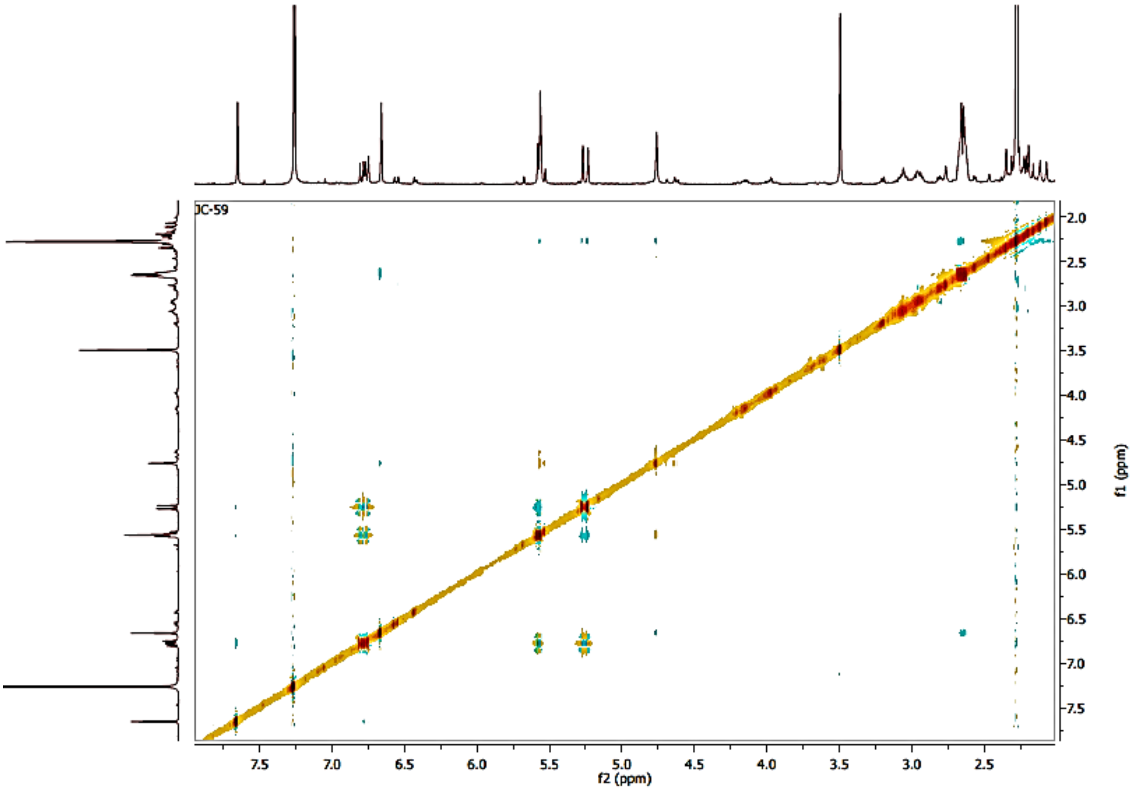

Figure S12. HMBC spectrum of compound **2**

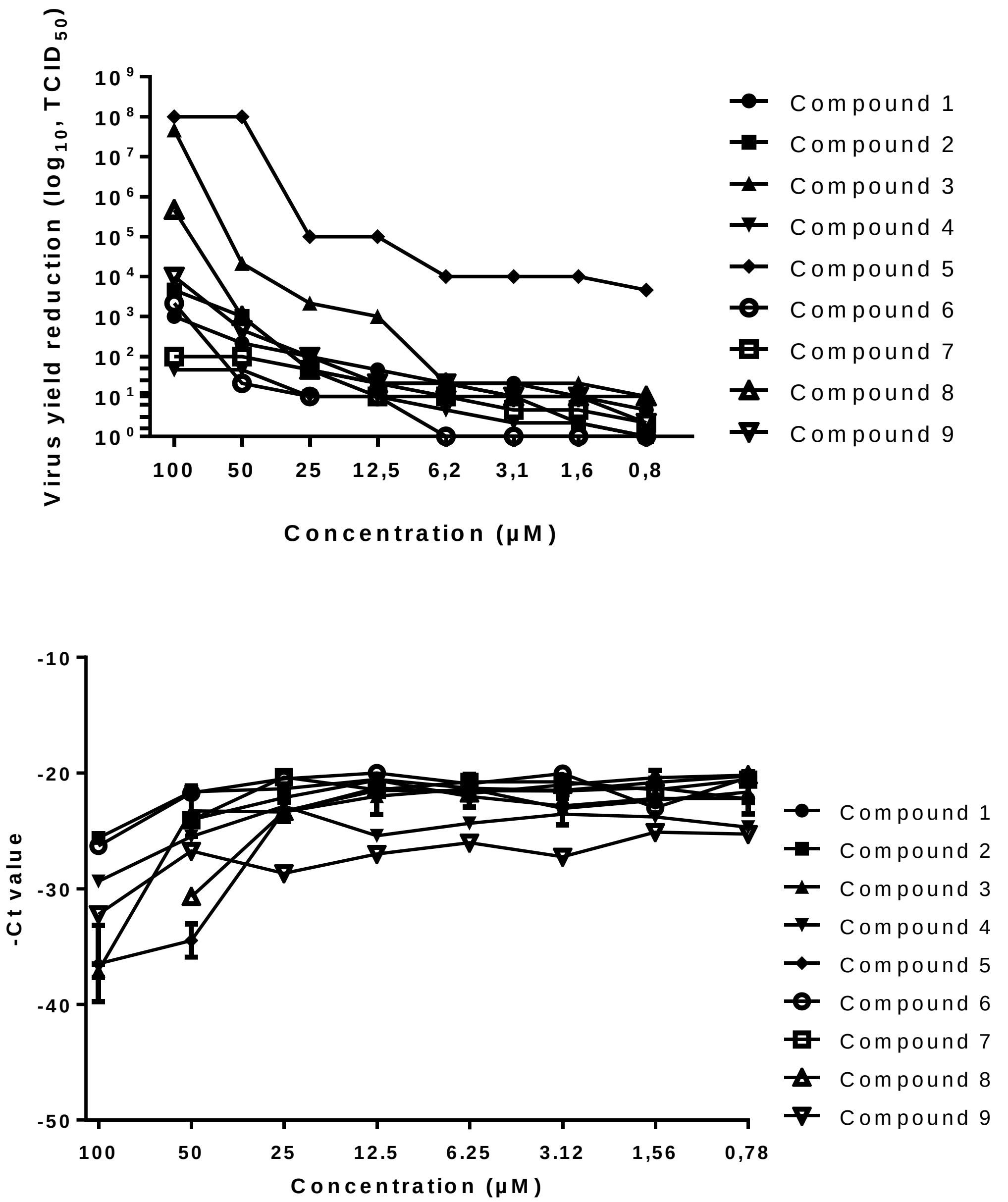

Figure S13. Antiviral effect of compounds **1**–**9**.

Vero cells were infected with HSV-2 (MOI 0.01) in the presence of various concentrations of different compounds for 24 h (n=4). At 24 h post infection, the cells were lysed and the virus yield reducing effect of the compounds was evaluated by comparing the yield to that seen on untreated Vero cells. The HSV-2 DNA concentration in the lysates was measured by direct qPCR (Data represent the average −Ct values +/− standard deviations).
